# Anthelmintic Activity of Crude Extract and Essential Oil of *Tanacetum vulgare* (Asteraceae) against Adult Worms of *Schistosoma mansoni*

**DOI:** 10.1155/2014/460342

**Published:** 2014-02-02

**Authors:** Loyana Silva Godinho, Lara Soares Aleixo de Carvalho, Clarissa Campos Barbosa de Castro, Mirna Meana Dias, Priscila de Faria Pinto, Antônio Eduardo Miller Crotti, Pedro Luiz Silva Pinto, Josué de Moraes, Ademar A. Da Silva Filho

**Affiliations:** ^1^Faculdade de Farmácia, Departamento de Ciências Farmacêuticas, Universidade Federal de Juiz de Fora, 36036-900 Juiz de Fora, MG, Brazil; ^2^Instituto de Ciências Biológicas, Departamento de Bioquímica, Universidade Federal de Juiz de Fora, 36036-900 Juiz de Fora, MG, Brazil; ^3^Departamento de Química, Faculdade de Filosofia, Ciências e Letras de Ribeirão Preto, Universidade de São Paulo, 14040-901 Ribeirão Preto, SP, Brazil; ^4^Núcleo de Enteroparasitas, Instituto Adolfo Lutz, 01246-902 São Paulo, SP, Brazil; ^5^Faculdade de Ciências de Guarulhos, FACIG/UNIESP, 07025-000 Guarulhos, SP, Brazil

## Abstract

Schistosomiasis, a parasitic disease caused by trematode flatworms of the genus *Schistosoma*, affects more than 200 million people worldwide, and its control is dependent on a single drug, praziquantel. *Tanacetum vulgare* (Asteraceae) is used in folk medicine as a vermifuge. This study aimed to investigate the *in vitro* schistosomicidal activity of the crude extract (TV) and the essential oil (TV-EO) from the aerial parts of *T. vulgare*. TV-EO was obtained by hydrodistillation and analyzed by GC/MS, which allowed the identification of **β**-thujone (84.13%) as the major constituent. TV and TV-EO, at 200 **μ**g/mL, decreased motor activity and caused 100% mortality of all adult worms. At 100 and 50 **μ**g/mL, only TV caused death of all adult worms, while TV-EO was inactive. TV (200 **μ**g/mL) was also able to reduce viability and decrease production of developed eggs. Confocal laser scanning microscopy showed morphological alterations in the tegument of the *S. mansoni* surface after incubation with TV (50 and 100 **μ**g/mL). Quantitative analysis on the schistosomes tegument showed that TV caused changes in the numbers of tubercles of *S. mansoni* male worms in a dose-dependent manner. The findings suggest that *T. vulgare* is a potential source of schistosomicidal compounds.

## 1. Introduction

Schistosomiasis, a parasitic disease caused by trematode flatworms of the genus *Schistosoma*, is the second major neglected tropical disease, with major economic and public-health consequences. The major etiological agent of human schistosomiasis is *Schistosoma mansoni*, and it is estimated that more than 200 million people are infected and 779 million people are at risk of infection worldwide [1, 2].

The treatment of schistosomiasis is based on the control of adult worms in infected patients, with praziquantel (PZQ) being the most widely used drug. However, PZQ does not prevent reinfection, is inactive against juvenile schistosomes, and has only a limited effect on already developed liver and spleen lesions [3, 4]. These limitations, in combination with a considerable concern about the development of PZQ resistance, have motivated the scientific community to develop novel and inexpensive drugs against schistosomiasis [5, 6]. In this regard, there has been intensification in the search for new schistosomicidal compounds from natural sources, mainly from plants, which continue to be a major source of biologically active metabolites that may provide lead structures for the development of new drugs [6, 7].

A number of recent studies have investigated the schistosomicidal activity of plants and their isolated compounds [6–8]. A number of extracts, essential oils, and isolated natural compounds displaying *in vitro* schistosomicidal activity have been identified [9–13]. Among tested plants, several members of the family Asteraceae have shown promising *in vitro* schistosomicidal activity [14, 15].

Plants of the genus *Tanacetum *(Asteraceae) are used over the years in folk medicines all over the world for many medicinal purposes, including anti-inflammatory and anthelmintic [16, 17]. *Tanacetum *extracts and its isolated compounds have been also reported to exhibit antiviral activity [17] and trypanocidal [18] and leishmanicidal [19] activities.

*Tanacetum vulgare *L., known as “Tansy” in Europe and “catinga-de-mulata” in Brazil, is widely used in folk medicine as a vermifuge and anti-inflammatory [16]. Also, the aerial parts of this plant are popularly used to treat migraine, neuralgia, and rheumatism, and as an anthelmintic and insect repellent [17]. Phytochemical studies have shown that *T. vulgare* contain several biologically active metabolites, mainly sesquiterpene lactones [16–19].

On the basis of the folkloric uses of *T. vulgare* as an anthelmintic and a vermifuge, as well as in continuation of our search for active natural sources against *S. mansoni* [20–24], the present study evaluated the *in vitro *schistosomicidal effects of the extract and the essential oil of *T. vulgare*, which have not yet been described.

## 2. Materials and Methods

### 2.1. Plant Material and Extraction

Aerial parts of *T. vulgare* L. were collected from the Horto Medicinal da Faculdade de Fármacia, in Juiz de Fora, MG, Brazil. A voucher specimen was deposited in the herbarium of the Botany Department of the Universidade Federal de Juiz de Fora, MG, Brazil. The plant material (229 g) was dried, powdered, and exhaustively extracted, by maceration at room temperature, using EtOH/H_2_O 8 : 2 (v/v). After filtration, the solvent was removed under reduced pressure to yield 5.3 g of the crude hydroalcoholic extract of *T. vulgare* (TV).

### 2.2. Essential Oil Extraction

The essential oil of *T. vulgare* (TV-EO) was obtained from aerial parts by hydrodistillation using a Clevenger-type apparatus for 4 h. After manual collection of the essential oil, traces of water were removed by freezing the sample below 4°C, followed by transfer of the unfrozen essential oil to a new vial. The yield was calculated as % (w/w) of the fresh aerial parts.

### 2.3. CG/MS Analysis

The TV-EO was analyzed by GC/MS analysis using a Shimadzu QP2010 Plus (Shimadzu Corporation, Kyoto, Japan) system equipped with an AOC-20i autosampler and a Restek Rtx-5MS fused-silica capillary column (5% phenyl-, 95% methylpolysiloxane; 30 m × 0.25 mm i.d., film thickness 0.25 *μ*m). The oven temperature was programmed to increase from 60 to 240°C at 3°C/min; injector temperature, 240°C; ion-source temperature, 280°C; carrier gas, He (1.0 mL/min); split ratio, 1 : 10; injection volume, 0.1 *μ*L. The mass spectrometer was operated in the electron ionization mode (70 eV), and the spectra were taken with a scan interval of 0.5 s over the mass range 40–600 Da. The quantification of each TV-EO constituent was done by internal normalization (%). The identification of the TV-EO components was based on the comparison of their retention indices (RI; determined in relation to the RT of *n*-alkanes (C_8_–C_26_)) and mass spectra with those of the *Wiley 7*, *NIST 08*, and *FFNSC 1.2* spectral libraries, as well as with those reported in the literature [25].

### 2.4. Parasite. *S. mansoni*

(BH strain Belo Horizonte, Brazil) worms have been maintained in *Biomphalaria glabrata* snails and *Mesocricetus auratus* hamsters hosts at the Adolfo Lutz Institute (São Paulo, Brazil). Female hamsters, weighting 20–22 g, were infected by subcutaneous injection of 150 cercariae. After 9 weeks, adults *S. mansoni* specimens were recovered from the hamster by perfusion with RPMI 1640 medium supplemented with heparin [26]. The worms were washed in RPMI 1640 medium (Gibco) supplemented with 200 *μ*g/mL of streptomycin, 200 UI/mL of penicillin (Invitrogen), and 25 mM of Hepes. Pairs of adult worms (male and female) were incubated in a 24-well culture plate (Techno Plastic Products, TPP) containing 2 mL of the same medium supplemented with 10% heat-inactivated calf serum at 37°C in a 5% CO_2_ atmosphere [27, 28]. All experiments were authorized by the Committee for Ethics in Animal Care of Adolfo Lutz Institute, in accordance with nationally and internationally accepted principles for laboratory animal use and care.

### 2.5. *In Vitro* Studies with *S. mansoni*

For the *in vitro* test with *S. mansoni*, TV and TV-EO were evaluated at concentrations of 10, 50, 100, and 200 *μ*g/mL, according to works previously described [29, 30]. Samples were dissolved in 0.5% DMSO and added to RPMI 1640 medium containing one pair of adult worms that had been allowed for 24 h to adapt to the culture medium. Worm motor activity, tegumental alterations, changes in the pairing, and survival of the parasites were monitored on daily basis for 3 days using an inverted microscope and a stereomicroscope (SMZ 1000, Nikon). All experiments were carried out in triplicate and repeated at least three times, using 10 *μ*M praziquantel (PZQ) as positive control group, and RPMI 1640 medium and RPMI 1640 with 0.5% DMSO as negative control groups.

### 2.6. Tegumental Changes

The quantification of the number of tubercles was performed for TV (the most active sample in tegument) using a confocal microscope. After the established times or in the occurrence of death, the parasites were fixed in formalin-acetic-alcohol solution (FAA) and analyzed under a confocal microscope (Laser Scanning Microscopy, LSM 510 META, Zeiss) at 488 nm (exciting) and 505 nm (emission) as described by [28, 29]. A minimum of three areas of the tegument of each parasite were assessed. The numbers of tubercles were counted in 20,000 *μ*m^2^ of area calculated with the Zeiss LSM Image Browser software. A blind analysis was performed by observer with experience and training in parasitology.

### 2.7. Viability Assay

For the viability assay against *S. mansoni*, pairs of adult worms were incubated for 120 h with TV (the most active sample) at concentration of 200 *μ*g/mL; the viability assay was performed according to MTT assay [31, 32]. After incubation, each pair of adult worms was placed individually into wells (96-well plates) containing 100 *μ*L of phosphate-buffered saline (PBS) with 5 mg MTT/mL for 30 min at 37°C. The solution was carefully removed and replaced with 200 *μ*L of DMSO and the worms were allowed to stand in DMSO at room temperature for 1 h. The absorbance was read at 550 nm using as negative control groups RPMI 1640 medium and RPMI 1640 with 0.5% DMSO. Heat-killed worms at 56°C were used as positive controls groups.

### 2.8. Statistical Analysis

The statistical tests were performed with the GRAPHPAD PRISM (version 4.0) software. Significant differences were determined by one-way analysis of variance (ANOVA) and applying Tukey's test for multiple comparisons with a level of significance set at *P* < 0.05.

## 3. Results and Discussion 

Many plants have been used throughout the world in traditional medicine for the treatment of parasite diseases [6]. In this scenario, several *in vitro* studies against *Schistosoma *species have been performed with crude plant extracts and essential oils, especially from species from the Asteraceae family [6, 14, 15]. *T. vulgare* is popularly used as anthelmintic and vermifuge, which encouraged us to evaluate the *in vitro* effect of its crude extract and essential oil against adult worms of* S. mansoni*, which have not been performed to date.

As shown in Table 1, positive control (PZQ, 10 *μ*M) resulted in the death of all parasites within 24 hours, whereas no mortality was observed in the worms of the negative (RPMI medium) and solvent control (RPMI medium plus 0.5% DMSO) groups. On the other hand, after 24 h of incubation, both TV and TV-EO, at 200 *μ*g/mL, cause 100% mortality of all adult worms. However, at 100 and 50 *μ*g/mL, only TV causes 100% mortality, while TV-EO was inactive at concentrations of 100 to 10 *μ*g/mL after 72 h of incubation (Table 1).

Also, all pairs of worms separated into individual male and female when exposed to 100 and 200 *μ*g/mL of both TV and TV-EO after 24 h of incubation. However, at 50 *μ*g/mL, 100% worm pairs were separated by TV-EO only after 72 h of incubation. Moreover, male and female adults showed a significant decrease in motor activity after 24 h of incubation with 200 *μ*g/mL of TV and TV-EO. However, motor activity was significantly decreased by the action of TV and TV-EO (at concentrations ranging from 50 to 100 *μ*g/mL) only after 72 h of incubation. PZQ (10 *μ*M) caused 100% mortality but no separation of worm pairs, whereas no effects were observed in worms in the negative (RPMI 1640 medium) and control (RPMI medium plus 0.5% DMSO) groups. Additionally, only TV (50, 100, and 200 *μ*g/mL) caused significant tegumental alterations in adult worms after 72 h of incubation (Table 1).

In relation to the decrease in motor activity, studies revealed that the musculature of *S. mansoni* can serve as a therapeutic target, because the motility of worms is associated with important neurotransmitters or neuromodulators [33]. Nematode neuropeptide systems comprise an exceptionally complex array of ~250 peptidic signaling molecules that operate within a structurally simple nervous system of ~300 neurons. Thus, these signaling systems can provide tools for the discovery of more amenable targets such as neuropeptide receptors or neuropeptide processing enzymes [34].

In recent years, some essential oils have been reported as promising schistosomicidal agents [12–15, 33]. In this study, the *in vitro* effects of different concentrations of the essential oil from the *T. vulgare* (TV-EO) on *S. mansoni* adult worms were assessed.

**Figure 1:**
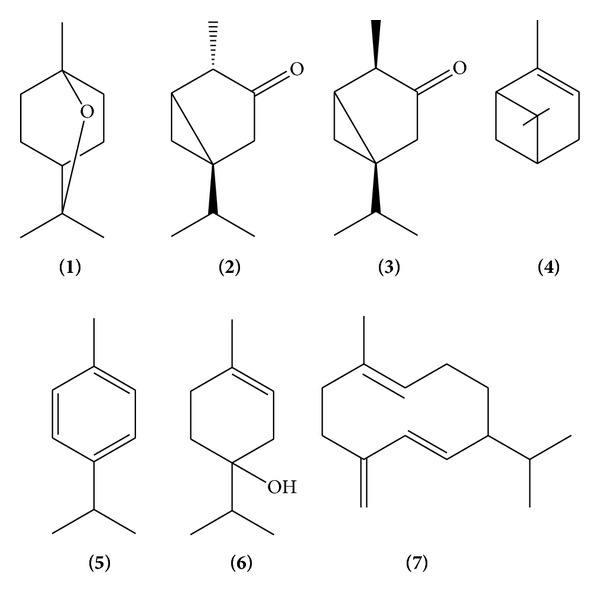
Chemical constituents identified by GC-MS in the essential oil of *T. vulgare *(TV-EO).

The essential oil of *T. vulgare* (TV-EO) was obtained by hydrodistillation of the aerial parts, yielding 0.05% (w/w). The chemical composition of this oil is shown in Table 2 and Figure 1. A total of 7 compounds were identified, being six monoterpenes (89.58%) and one sesquiterpene (6.81%). **β**-Thujone (**3**, 84.15%) was identified as the major constituent (Figure 1) of TV-EO. **β**-Thujone has also been previously described as major constituent of the essential oils obtained from specimens of *T. vulgare* collected in Eurasian and North America [35]. Considering the chemical composition of TV-EO, its schistosomicidal *in vitro *activity may be related to **β**-thujone, its major constituent. Studies have associated the anthelmintic activity of the essential oils obtained from specimens of *T. vulgare* with **β**-thujone [36]. It has been reported that thujone may act on GABA_A_ receptor, similarly to some anthelmintic drugs, such as ivermectin [37, 38]. Also, considering the mechanism by which TV-EO exerts its *in vitro* schistosomicidal effect, essential oils may have no specific cellular target, because lipophilic compounds, typically present in essential oils, may pass through the cell wall, tegument, and cytoplasmic membrane, damaging their structures, which may lead to the cellular lysis [39].

According to results observed in preliminary survival of 56-day-old adult worms of *S. mansoni* test (Table 1), TV was more active than TV-EO, due to its ability to cause 100% mortality of all parasites at lower concentrations, as well as to cause significant tegumental alterations. Because of that, TV was further tested to assess viability, oviposition, and morphological alterations in the parasite's tegument.

**Figure 2:**
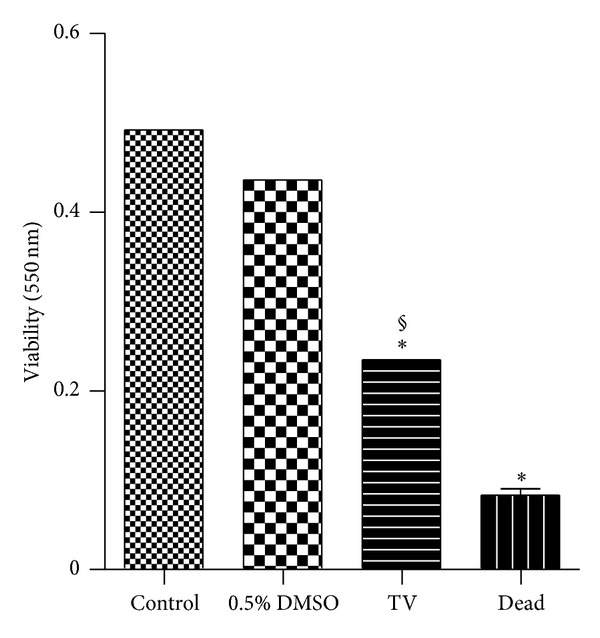
*In vitro *effect of the crude hydroalcoholic extract of *T. vulgare* (TV) on the viability of the *S. mansoni* adult worms. Pairs of adult worms were treated with TV at 200 *μ*g/mL during 120 h and the viability was measured by MTT assay at 550 nm. RPMI 1640 medium and 0.5% DMSO in RPMI 1640 medium were used as negative control groups. Heat-killed worms at 56°C (dead) were used as positive control group. The viability was expressed as mean of the absorbance values from three experiments. *Significantly different from control (*P* < 0.05);  ^§^significantly different from dead group (*P* < 0.05).

The viability of adult *S. mansoni* worms was evaluated during their *in vitro* incubation with TV (200 *μ*g/mL) after a period of 120 hours (Figure 2). A 50% reduction was observed in the worms viability incubated with TV when compared with the negative control groups. However, the viability of these worms was significantly higher than that of the group of worms dead by heating.

**Figure 3:**
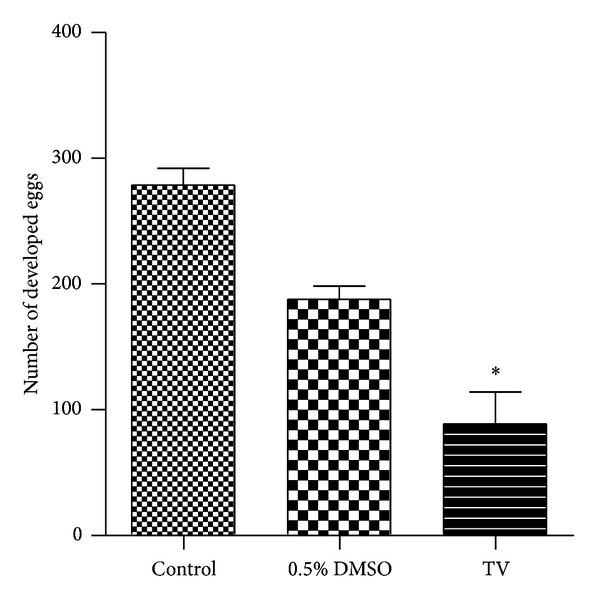
*In vitro *effect of the crude hydroalcoholic extract of *T. vulgare* (TV) at 200 *μ*g/mL on egg development (quantitative analysis of the development phenotype). After treatment, the eggs were microscopically examined and scored as developed or undeveloped based on the presence or absence of the miracidium. Data are presented as the mean of developed eggs from two separate experiments. *Significantly different from control (*P* < 0.05).

Regarding production of developed eggs by adult worms of *S. mansoni* (Figure 3), TV (200 *μ*g/mL) showed a significant decrease in the number of developed eggs, by 29.8%, after 120 h of incubation, in comparison with the negative control group treated with RPMI 1640 medium. However, the effect of TV on egg production may be correlated with its ability to separate adult worms into male and female.

The presence of *S. mansoni* eggs in the host tissues has been reported to be closely related to the pathology of human schistosomiasis [12, 20]. The permanent pairing of the schistosomes couples in the blood system of their hosts vertebrates throughout their lifespan causes high rate of oviposition, which is responsible for the resulting immunopathological lesions, characterized by inflammation and fibrosis in the target [13].

**Figure 4:**
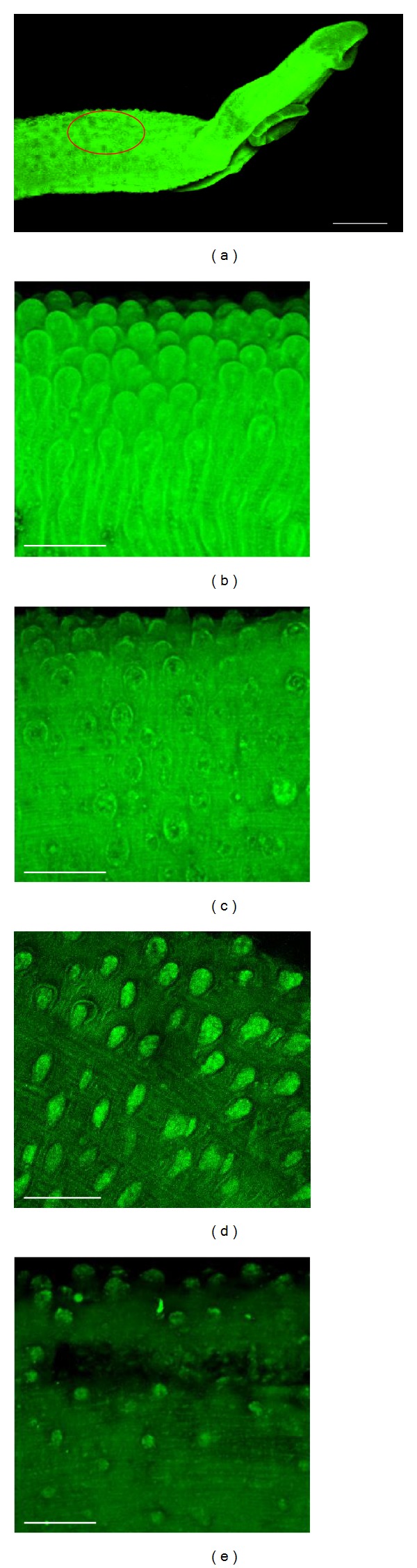
Confocal laser scanning microscopy investigation of the crude hydroalcoholic extract of *T. vulgare* (TV) *in vitro* schistosomicidal effect. In these experiments, pairs of adult worms were incubated in 24-well culture plates containing RPMI 1640 medium and treated with different concentrations of TV. After 120 h of incubation or in the case of death, adult male worms were fixed in FAA solution and fluorescent images were obtained using confocal microscopy (Carl Zeiss LSM 510 META). General view of the anterior worm region showing, in red, the location where tegument was analysed (a). Control worms in RPMI 1640 with 0.5% DMSO (b), and 10 *μ*M praziquantel (c), compared to worms treated with 50 *μ*g/mL of TV (d), and 100 *μ*g/mL of TV (e). Scale bars, 500 *μ*m (a) and 50 *μ*m (b–e).

Moreover, light microscopic investigations (Table 1) showed that TV caused morphological alterations in the parasite's tegument, with no distinction between male and female worms (data not shown). No tegumental changes in adult worms were observed for the negative control group, while the positive control (PZQ) had tegumental alteration in 100% of the worms. In addition, the effect of TV on the parasite's tegument was monitored microscopically using confocal laser scanning microscopy. As shown in Figure 4, morphological alterations of the tegument on the *S. mansoni *surface were detected with TV at 50 *μ*g/mL (Figure 4(d)) and 100 *μ*g/mL (Figure 4(e)). Meanwhile, no abnormality was seen in the worms maintained in the negative control group. Thus, a pattern consisting of a combination of changes in the surface morphology was detected and correlated to the death of the adult worms. These pronounced changes in the aspect of the tubercles, which often appeared collapsed and disrupted, were similar to those reported in studies with some isolated natural compounds, such as piplartine, epiisopiloturine, and (+)-limonene epoxide [29, 30, 40].

**Figure 5:**
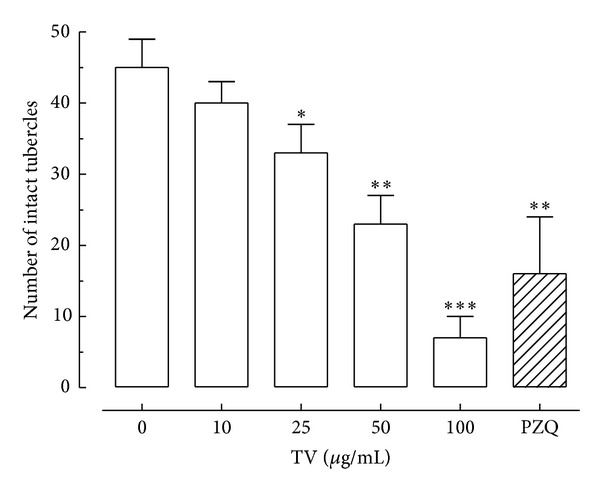
Quantitative analysis of morphological alterations on the tegument of *S. mansoni* after exposure to different concentrations of the crude hydroalcoholic extract of *T. vulgare* (TV). The quantification of the number of tubercles was performed using three-dimensional images obtained from laser scanning confocal microscopy. Indicated are numbers of intact tubercles, and these numbers were measured in a 20 000 *μ*m^2^ area in a dorsal region of *Schistosoma mansoni* adult male worm (see Figure 4(a)) and calculated with the Zeiss LSM Image Browser software. Praziquantel (PZQ, 10 *μ*M) was used as a positive control. A minimum of three tegument areas of each parasite were assessed. Values are means ± SD (bars) of ten male adult worms. *P < 0.05, **P < 0.01, and ***P < 0.001 compared with untreated group.

Additionally, morphological alterations on *S. mansoni* tegument were evaluated quantitatively after exposure to different concentrations of TV. In this quantitative analysis, areas of tegument of male worms were assessed, and the numbers of tubercles on the dorsal surface of parasites were counted [23, 27–29]. As shown in Figure 5, TV caused changes on tubercles of *S. mansoni* male worms in a dose-dependent manner. For example, the number of intact tubercles in an area of 20 000 *μ*m^2^ on male worms of the negative control was 46, while in the groups exposed to 10, 25, 50, and 100 *μ*g/mL of TV the number was, respectively, 39, 32, 23, and 16. Similar results, with the pattern of tubercle destruction in a dose-dependent manner, were obtained from the paired schistosomes exposed to some natural products, such as antimicrobial peptide dermaseptin and (+)-limonene epoxide [23, 29].

The tegument is extremely important to the infection success and survival in the host, and it has been a major target for the development of antischistosomal drugs [28], since most of the currently used drugs against schistosomes, such as PZQ [41], mefloquine [42], and artemether [43], act by damaging the schistosome tegument.

Several compounds, mainly sesquiterpene lactones (STL), have been identified as active constituents in previous phytochemical studies of the aerial parts of *T. vulgare *[17–19]. Some STL from *T. vulgare*, mainly parthenolide, proved to be active against some parasites, such as *Trypanosoma cruzi* [18] and *Leishmania amazonensis *[19]. Also, *T. vulgare *shows remarkable antioxidant properties, mainly due to its phenolic compounds content, especially flavonoids and caffeoylquinic acid derivatives [44]. The wide spectrum of *T. vulgare* activities can be mainly ascribed to the occurrence of STL [16]. Some STL have been reported as molluscicidal compounds, showing activity against adult *Biomphalaria *sp., a snail directly implicated in the transmission of schistosomiasis [45].

The mechanism by which TV exerts its *in vitro* schistosomicidal effect is unclear. However, adult worms of *S. mansoni* died accompanied by destruction of the worm body, and a relationship between tegumental damage and the death of worms was observed in the *in vitro* assays. Furthermore, *in vivo* investigations in mice infected by *S. mansoni* are necessary to determine the clinical potential of TV to treat schistosomiasis. In this case, mice may be treated orally using single or multiple oral doses at different life-cycle stages (e.g., schistosomula, juvenile, and adult worms). In addition, toxicological studies (e.g., acute oral LD_50_ of TV) should be examined.

## 4. Conclusion

In this study, we have reported an investigation on the *in vitro* schistosomicidal effects of the crude hydroalcoholic extract (TV) and the essential oil from *T. vulgare* (TV-EO) for the first time. It was demonstrated that both TV and TV-EO possess significant activity against adult worms of *S. mansoni*. The activity of TV-EO may be related, at least in part, to monoterpene thujones, which were detected as major constituents in TV-EO. Our study reinforced the traditional use of *T. vulgare* as a vermifuge and anthelmintic. Considering the obtained results, further biological studies, as well as phytochemical investigations, are in progress with TV in order to identify its major active compound and elucidate its mechanism(s) of schistosomicidal action.

## Figures and Tables

**Table 1:** *In vitro* effects of crude hydroalcoholic extract (TV) and essential oil of *T. vulgare* (TV-EO) against *S. mansoni *adult worms.

Groups	Incubation period (h)	Separated worms (%)^a^	Dead worms (%)^a^	Decrease in motor activity (%)^a^		Worms with tegumental alteration (%)^a^	
				Slight	Significant	Partial	Extensive
Control^b^	24	0	0	0	0	0	0
	72	0	0	0	0	0	0
0.5% DMSO	24	0	0	0	0	0	0
	72	0	0	0	0	0	0
PZQ^c^	24	0	100	0	100	0	100
	72	0	100	0	100	0	100
TV							
10 *μ*g/mL	24	0	0	0	0	0	0
	72	0	0	0	0	0	0
50 *μ*g/mL	24	0	0	0	0	0	0
	72	0	100	0	100	0	100
100 *μ*g/mL	24	0	0	0	0	0	0
	72	100	100	0	100	100	100
200 *μ*g/mL	24	0	100	0	100	0	100
	72	100	100	0	100	0	100
TV-EO							
10 *μ*g/mL	24	0	0	0	0	0	0
	72	50	0	0	100	0	0
50 *μ*g/mL	24	0	0	0	0	0	0
	72	100	0	0	100	0	0
100 *μ*g/mL	24	100	0	0	0	0	0
	72	100	0	0	100	0	0
200 *μ*g/mL	24	100	100	0	100	0	0
	72	100	100	0	100	0	0

^a^Percentages relative to the 20 worms investigated. ^b^RPMI 1640. ^c^Tested at concentration of 10 *μ*M.

**Table 2:** Composition of the essential oil of *T. vulgare* (TV-EO) identified by CG/MS analysis.

Compounds	RI_exp⁡_^a^	RI_lit_^b^	Content (%)^c^	Identification^d^
1,8-Cineol (**1**)	1036	1031	0.55	RI, MS
*α*-Thujone (**2**)	927	930	1.64	RI, MS
*β*-Thujone (**3**)	1117	1114	84.13	RI, MS
*α*-Pinene (**4**)	935	939	1.04	RI, MS
*p*-Cimene (**5**)	1025	1026	0.68	RI, MS
Terpinen-4-ol (**6**)	1167	1171	1.54	RI, MS
Germacrene-D (**7**)	1480	1480	6.81	RI, MS
Not identified	1499	—	1.12	RI, MS
Not identified	1965	—	1.68	RI, MS
Total			**96.39**	

Monoterpenes: 89.58%				
Sesquiterpenes: 6.81%				

^a^RI_exp_: retention index determined relative to *n*-alkanes (C_8_–C_26_) on the Rtx-5MS column. ^b^RI_lit_: retention index from [25]. ^c^Calculated from the peak area relative to the total peak area. ^d^Compound identification: RI, comparison of the RI with those of [25]; MS: comparison of the mass spectra with those of the Wiley 7, NIST 08, and FFNSC 1.2 spectral libraries as well as with those of [25].
